# Supplementary material for: Contribution to Characterizing the Meat Quality of Protected Designation of Origin Serrana and Preta de Montesinho Kids Using the Near-Infrared Reflectance Methodology
Source: Foods. 2024 May 19;13(10):1581. doi: 10.3390/foods13101581 (PMC11121219; doi:10.3390/foods13101581)
Supplement: Supplementary file 1 [file foods-13-01581-s001.zip › foods-2997733-supplementary.pdf]

Supplementary Material

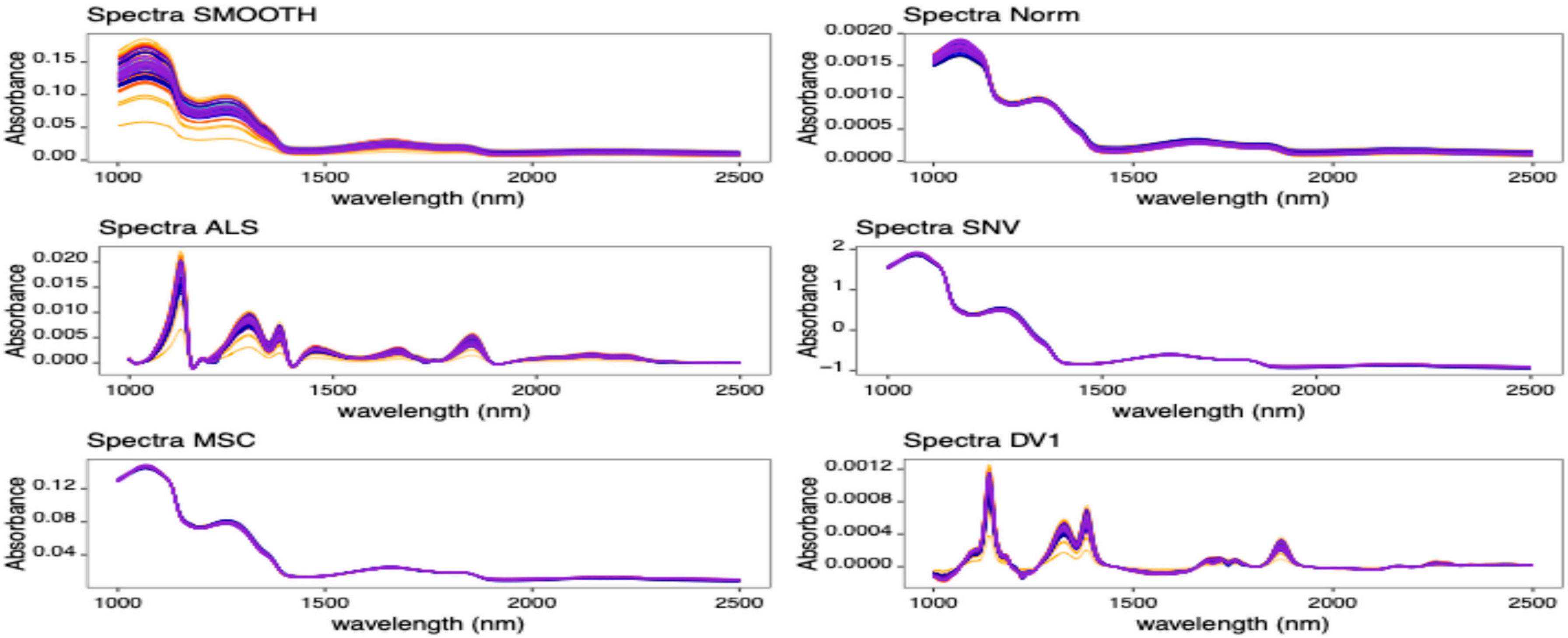

Figure S1. Representation of SMOOTH, Norm, ALS, SNV, MSC, DV1, DV2, Norm+DV1, ALS+DV1, SNV+DV1, MSC+DV1 spectra obtained from the muscle samples.

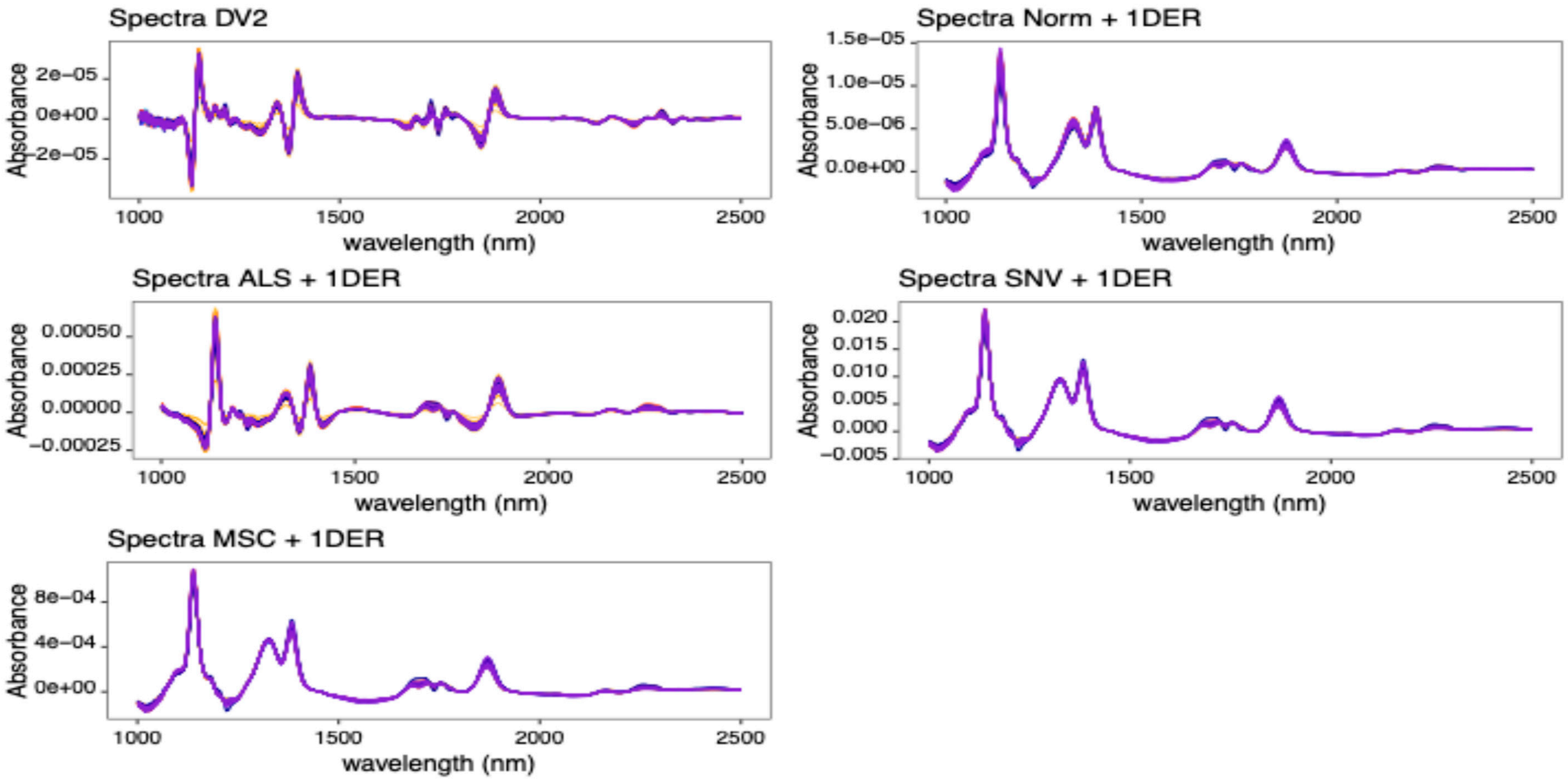

Figure S1. (continuation). Representation of all smoothed, NORM-SMT, ALS-SMT, SNV-SMT, MSC-SMT, DV1 and DV2 spectra obtained from the muscle samples.
